# Human milk composition and infant anthropometrics: overview of a systematic review with clinical and research implications

**DOI:** 10.1186/s13006-024-00652-x

**Published:** 2024-06-28

**Authors:** Meghan B. Azad, Meredith M. Brockway, Sarah M. Reyes

**Affiliations:** 1 Department of Pediatrics and Child Health, University of Manitoba, 501G-715 McDermot Avenue, Winnipeg, MB R3E 3P4 Canada; 2 Manitoba Interdisciplinary Lactation Centre (MILC), Children’s Hospital Research Institute of Manitoba, Winnipeg, Canada; 3 Faculty of Nursing, University of Calgary, Calgary, Canada; 4 Rev Bioscience, LLC, Boise, USA

**Keywords:** Breastfeeding, Human milk, Infant growth, Macronutrients, Micronutrients

## Abstract

**Background:**

Despite global public health organizations endorsing breastfeeding or human milk (HM) as the optimal source of nutrition for infants, detailed knowledge of how HM composition influences infant growth is lacking. In this commentary we summarize and interpret the key findings of a large systematic review on HM components and child growth (N = 141 articles included). We highlight the most consistent associations, discuss study quality issues, explore socio-economic and time trends in this body of research, and identify gaps and future research directions.

**Key Findings of Systematic Review:**

We grouped HM components into three categories: micronutrients (28 articles), macronutrients (57 articles), and bioactives (75 articles). Overall, we struggled to find consistent associations between HM components and infant growth. The majority of studies (85%) were of moderate or low-quality, with inconsistent HM collection and analysis strategies being identified as the most substantial quality concerns. Additional quality issues included failing to account for potential confounding by factors such as breastfeeding exclusivity and maternal body mass index.

**Considerations for Future Human Milk Research:**

Many opportunities exist for the future of HM research. Using untargeted metabolomics will expand our understanding of HM components beyond previously defined and well-understood components. Machine learning will allow researchers to investigate HM as an integrated system, rather than a collection of individual components. Future research on HM composition should incorporate evidence-based HM sampling strategies to encompass circadian variation as well as infant consumption. Additionally, researchers need to focus on developing high quality growth data using consistent growth metrics and definitions. Building multidisciplinary research teams will help to ensure that outcomes are meaningful and clinically relevant.

**Conclusion:**

Despite a large body of literature, there is limited quality evidence on the relationship between HM composition and infant growth. Future research should engage in more accurate collection of breastfeeding data, use standardized HM collection strategies and employ assays that are validated for HM. By systematically evaluating the existing literature and identifying gaps in existing research methods and practice, we hope to inspire standardized methods and reporting guidelines to support robust strategies for examining relationships between HM composition and child growth.

## Background

**Fig. 1:**
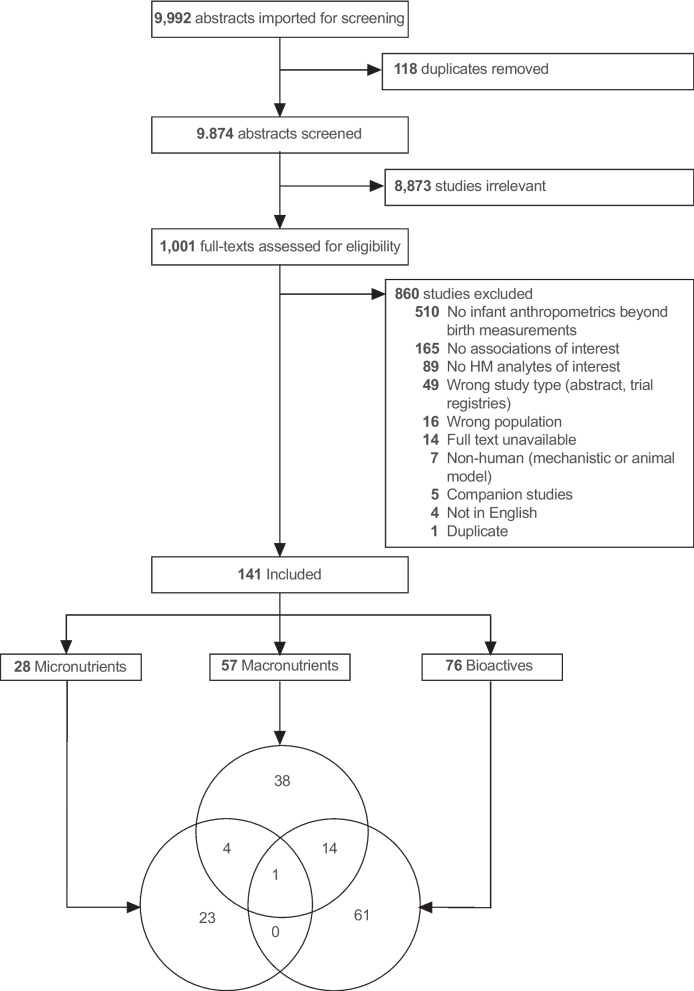
Systematic review of associations between human milk (HM) micronutrients and infant growth in the first 2 years: PRISMA flow diagram. Reasons for study exclusion were recorded in the order listed in the figure. Though some studies had more than one reason for exclusion, each study was only counted once (e.g., if a study reported no HM analytes of interest and was not in English, it was recorded as the former)

Recently our team from the International Milk Composition Consortium completed one of the largest systematic reviews to date examining the association between human milk (HM) components and growth in full-term infants through the second year of life. We screened 9,992 abstracts and 1001 full-text articles, and ultimately included 141 articles in this review **(**Fig. 1**)**. Due to the large number of articles included, we grouped the HM components into three categories and reported each in a separate manuscript: micronutrients (data extracted from 28 articles) [1], macronutrients (57 articles) [2], and bioactives (75 articles) [3].

We identified several recurring themes across all three manuscripts. These included study quality issues related to milk sampling times and strategies, use of unvalidated analytic techniques, inconsistent reporting of HM analytes and child growth outcomes, and studying HM components in a reductionist manner without examining how they interact as a system. We noted publication trends according to socioeconomic settings—presumably linked to research priorities, such as targeted funding for micronutrient research in low and middle income countries. We also observed trends over time according to the evolution of HM analysis technologies—such as the advent of mid-infrared HM analysers, which quickly became the dominant technique for HM macronutrient analysis in high-income settings. In this commentary we summarize our key findings and discuss these themes in-depth, highlighting how they impact the quality and applicability of evidence around HM components and child health. Finally, we suggest considerations for future research in the field of HM and lactation science.

## Key study findings: human milk and child growth

Overall, from the available data in the 141 included articles, we struggled to identify consistent associations between most individual HM components and infant growth. Among 16 micronutrients evaluated, iodine, manganese, calcium, and zinc concentrations were positively associated with several outcomes, while magnesium was negatively associated with linear growth during early lactation [1]. Specifically, HM iodine was linked with weight-for-length z-score (WLZ) but not with weight, length, or head circumference. Meanwhile, HM manganese concentration showed a positive association with weight-for-age z-score (WAZ) during early lactation, with no evidence of association with length or head circumference. Calcium *intake* from HM, rather than the concentration of calcium in HM, emerged as a significant predictor of infant weight and length gain. Overall, the findings were varied but suggested that zinc intake from HM may impact both weight, particularly in early infancy and when mothers are well-nourished, and length, especially in later infancy.

Assessment of the macronutrient literature revealed positive associations between HM protein and infant length, but not weight [2]. In general, HM carbohydrates were positively associated with infant weight. We did not observe any consistent associations between HM fat and infant growth—although major limitations were identified for fat analysis (described below). A few associations were identified for individual HM fatty acids and amino acids, though none had been replicated in more than a single study.

Among dozens of HM bioactive components investigated, the hormones leptin and adiponectin showed consistent inverse associations with infant growth [3]. No consistent associations were found between HM oligosaccharides and infant growth outcomes, although this is a relatively newer field of research. Interleukin-6 (IL-6) was the only immunomodulatory component in HM that had consistent associations with infant growth, demonstrating an inverse relationship with infant weight.

## Considerations arising

### Study quality issues

Over 85% of the 141 articles included in our review were considered moderate or low quality. Study quality was assessed using a modified Newcastle–Ottawa Scale developed in collaboration with subject matter experts [4] and evaluated across three domains: *HM exposure* (HM intake, HM collection techniques, laboratory sampling preparation techniques, and analytical method used – with ideal methods pre-specified for each HM component); *confounders considered* (e.g., breastfeeding exclusivity, adjustment for relevant maternal and infant characteristics); and *infant anthropometric assessments* (protocol and timing of measurements). Detailed quality assessment methods and results are available as supplementary material with each published manuscript [1–3].

#### Human milk exposure

A common quality issue related to the exposure assessment (i.e. HM collection and analysis) was that the time of day that HM was collected varied greatly between women, or was not recorded. This sampling strategy introduces “noise” that may obscure important associations with infant growth because some milk components vary in concentration diurnally, especially those related to the fat fraction of milk [5]. Similarly, milk collection practices (e.g., full or partial expression) varied across studies and were not always appropriate for the milk component investigated. For example, most studies measured total fat in milk using only a partial milk expression, which can give inaccurate results because fat concentrations vary throughout the course of each feeding [6]. Other common issues observed across studies included incomplete reporting of HM collection strategies and lack of accuracy and precision estimates related to analytical methods used to measure milk components.

#### Confounders considered

The most common quality issue across studies was failing to adequately account for confounding variables, including breastfeeding exclusivity and maternal body mass index. Several studies lacked details about complementary feeding or formula supplementation, even when follow-up extended past 6 months postpartum (i.e. beyond the recommended period of exclusive breastfeeding). These are important omissions because breastfeeding exclusivity and maternal body composition influence milk composition and production, affecting the availability of many HM components to the infant [7]. Both should be accounted for through study design or statistical analysis. We also noted inconsistencies in the definition of exclusive breastfeeding and suggest the research community adopts standard language such as those defined by Labbock or the WHO [8, 9].

#### Infant anthropometric assessments

We encountered extensive variation in the anthropometric outcomes measured across studies in this review. While most studies examined length and weight, less than 50% reported more clinically meaningful measures (e.g., body mass index, weight-for-length, weight-for-age, or length-for-age Z scores) and very few reported on longitudinal trajectories. Over 20 different anthropometric measures were reported across studies, which made it challenging to synthesize results and prohibited us from conducting meta-analyses. Reliability and reproducibility have long plagued infant growth research [10] and our findings demonstrate that this issue persists with extensive variation even among many studies published within the last 5 years.

Moving forward, researchers should 1) identify meaningful growth outcomes that act as good indicators of mortality and morbidity risk, 2) adhere to internationally recognized reference data sets for determining growth status such as the World Health Organization (WHO) [11] or INTERGROWTH [12], and 3) consider examining infant growth as a trajectory rather than a one-time measurement to account for infant growth velocity, rather than individual stature differences [13]. Reporting change scores, such as Z-score change rather than Z-score alone, is recommended to evaluate the effect of nutrition interventions on growth [13]. Adhering to these recommendations will help to align infant growth outcomes in a more meaningful way so that researchers and policy makers can develop clear and consistent guidelines from which clinicians can inform their practice.

### Time trends and socio-economic context

**Fig. 2:**
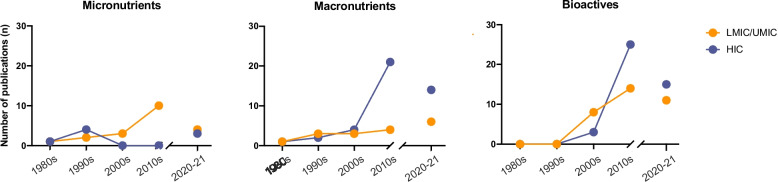
Distribution of publications included in our systematic review of HM composition and infant growth, by decade and income setting. LMIC, low middle income countries; UMIC, upper middle income countries; HIC, high income countries

Figure 2 demonstrates interesting trends in research on HM composition and infant growth over time and across income settings. Both micro and macronutrients in HM have been examined since the 1980’s. However, emphasis on micronutrient research shifted exclusively to low middle income country (LMIC) settings during the first 20 years of the twenty-first century. This is likely due to the priorities placed on solving micronutrient malnutrition which was highlighted as a significant issue in the late twentieth century. Over the early 1990s, repeated calls-to-action were issued to combat micronutrient malnutrition in lower resourced settings [14].

Research on macronutrient content in HM and infant growth has steadily been increasing over the past five decades, with emphasis shifting to research in high income country (HIC) settings. This may be due to the increasing accessibility of assays or milk analysers to measure macronutrients in HM, especially in HIC settings.

Most notably, prior to the 2000’s, bioactives in HM were not studied. This is likely due to a lack of awareness or priority placed on these components, as well as inadequate assays to study them. The recent increase in research on HM bioactivity was preceded by a national breastfeeding campaign launched in the US in the early 2000’s and a push for science to better understand the biological differentiation of HM from formula [15]. Notably, bioactive components have been studied more frequently in HIC than LMIC settings, potentially due to the high cost of measuring these components.

Research efforts have increased for all HM component types in recent years, with the number of publications in 2020–21 already approaching the totals for the entire preceding decade. This is likely to intensify even further with new targeted funding calls [16, 17] and global initiatives focused on HM [18, 19]. However, with the exception of micronutrient research, HM research is dominated by studies conducted in HIC settings. Emphasis should be placed on prioritizing funding to enhance global representation of all countries in HM science to ensure that research from HIC and LMIC settings is equally supported. Further, research conducted in LMIC settings should be meaningfully co-designed and co-conducted with local researchers and stakeholders [20].

### Leveraging existing datasets

Notably, 165 studies were excluded from our review because although they reported on HM analytes and infant anthropometrics, they did not investigate their associations. Collectively, this represents a tremendous amount of data and a huge missed opportunity to address an important knowledge gap on this subject. For studies where these valuable data remain accessible and resources are available (or could be obtained) to re-analyze them, it would be informative and cost-efficient to leverage existing datasets for producing new evidence on the association of HM and infant growth.

### The future of HM research

Our understanding of HM composition is expanding at an incredible rate. Gone are the days when we viewed HM as simply a source of nutrition for the infant - we now understand that HM is a complex matrix, perhaps even a “living tissue” [21], containing many interrelated components that work both independently and synergistically to support infant growth and development [22]. Nevertheless, we still have a long way to go before we can fully define and understand the composition of HM and its impact on the growing infant.

The advent of untargeted metabolomics is allowing researchers to identify new compounds in HM [23], the actions of which are still unknown. Much of the evidence included in this review is focussed on previously identified and relatively well understood components. However, two studies used untargeted metabolomics: Isganaitis et al., [24] and Wu et al. [25] both found relationships between previously unnamed HM components and infant anthropometrics, highlighting the value of applying new technologies to identify novel components in HM that may influence infant growth and development.

Implementing ‘omic’ strategies (e.g. metabolomics, proteomics, microbiomics) for HM research will generate large amounts of data that require innovative methods for analysis. Machine learning approaches allow researchers to create prediction and classification models from large amounts of data that can integrate information from multiple domains [26, 27]. Using machine learning strategies will allow researchers to investigate HM as a system, moving beyond traditional reductionist approaches [7]. Notably, in order to develop meaningful results using machine learning strategies, it is essential that research teams include expertise in all relevant domains (i.e. machine learning, infant nutrition, HM components and clinical outcomes of interest).

It is important to recognize that new ‘omic technologies and machine learning approaches cannot compensate for inadequate HM sampling strategies or poorly validated HM analytic techniques. It remains critical that both HM and infant growth data are high quality and consistently defined [26], and that HM collection strategies are thoughtfully designed to accurately reflect infant consumption. While global standards exist for collecting and reporting infant growth data [11, 12], there are no comparable standards for HM research. Our review highlights a need and opportunity to develop standardized approaches and guidelines for human milk research, perhaps drawing inspiration from other specialized analytical fields such as the STORMS checklist for human microbiome studies [28]. In parallel, it will be important to prioritize improving the quality and accessibility of assays and instruments for HM analytics.

## Conclusion

Overall, this review highlights that despite the relatively large evidence base, we have a relatively limited understanding of the relationships between HM composition and infant growth. By highlighting key knowledge gaps, emerging techniques, limitations of existing studies and opportunities for future research, we aim to inspire enhanced research strategies that will produce accurate, equitable, and meaningful evidence. Based on our findings, we recommend **(**Table 1**)** that HM researchers focus on: 1) accurate collection of breastfeeding data (exclusivity and feeding mode),  2) using appropriate and standardized HM collection strategies [29], 3) using and developing assays that are validated for HM, 4) applying statistical approaches to evaluate HM as a system,  5) measuring infant outcomes that are universal and meaningful to clinicians, and 6) collaborating with research partners globally to enhance diversity and representativeness. Additionally, we recommend investing in the development of standardized methodological and reporting guidelines for HM research. Collectively, these strategies will support high quality evidence to advance the field of HM science and spotlight this discipline as a priority for governments, healthcare organizations, and research funding agencies on a global scale.

**Table 1:** Priorities for Human Milk (HM) Research

Priority Areas
• Accurate collection of breastfeeding data (exclusivity and feeding mode)
• Standardized HM collection strategies appropriate for the analyte(s) of interest
• Standardized collection, calculation, and reporting of infant anthropometric assessments
• Developing and validating assays for HM
• Applying statistical approaches to evaluate HM as a system
• Collaboration with research partners globally to enhance diversity and representativeness
• Infant outcomes that are universal and meaningful to clinicians

## Data Availability

No datasets were generated or analysed during the current study.
